# The case of a 51‐year‐old man with 15 years of progressive leg spasticity

**DOI:** 10.1002/acn3.51380

**Published:** 2021-05-11

**Authors:** Danielle N. Larson

**Affiliations:** ^1^ Northwestern University Parkinson’s Disease and Movement Disorders Center Chicago Illinois USA

## Summary of Case

The patient is a 51‐year‐old male with 15 years of progressive leg weakness and inability to walk. At age 34, after hospitalization for a chemical fire as a firefighter, his gait changed that he would lean to the left. This slowly progressed over the years with increased stiffness and proximal leg weakness, requiring a cane since age 49. He rarely falls. He has no upper extremity weakness and no sensory changes. He denies bowel problems, but for the past several years he had urinary urgency and mild incontinence, for which he takes oxybutynin.

He had a baclofen pump placed at age 39 with some improvement in leg stiffness, but his symptoms have progressed over the past 2–3 years with decreased ability to walk long distances. He recently was started on tizanidine without benefit. His family history is only remarkable for a brother with “neuropathy” and gait difficulties.

On exam, mental status and cranial nerve evaluation is normal. His upper extremity strength, tone, and reflex testing is normal, but bilateral lower extremity strength is as follows: 5‐/5 hip flexion, 5/5 knee flexion and extension, 5‐/5 dorsiflexion, 5/5 plantar flexion, with marked increased tone and brisk, 4+ patellar reflexes, non‐sustained ankle clonus, and upgoing toes.

In workup, the following lab studies were unremarkable: ANA, creatine kinase, ESR, CRP, B12, ANA and auto‐immune panel, and CSF analysis. MRI Brain, MRI Cervical and Lumbar Spine, and EMG/NCS were normal. Genetic testing, GeneDx “Comprehensive Hereditary Spastic Paraplegia Panel” of next‐generation sequencing and deletion/duplication analysis of 36 genes, revealed the patient to be compound heterozygous for a known pathogenic variant and a likely pathogenic variant mutation in the SPG7 gene, each on separate alleles.

### Diagnosis

Hereditary Spastic Paraplegia Type 7.

### Take‐home points

- HSP should be considered on the differential for the causes of progressive difficulty walking with bilateral lower extremity spasticity, hyperreflexia, and weakness, particularly when imaging is unrevealing.^1^
- There are “complicated” phenotypes of HSP that include extra‐neurologic symptoms such as cerebellar ataxia, peripheral neuropathy, and optic neuropathy.^2^
- Patients presenting with an HSP phenotype must have spinal imaging to rule out intramedullary spinal cord pathology or dural AV fistula.
- Initial genetic testing of limited known HSP genetic variants may not be sufficient to identify pathologic variants, and in these cases, more detailed genetic testing should be considered.
